# Future Climate Change Increases the Risk of Suitable Habitats for the Invasive Macrophyte *Elodea nuttallii*

**DOI:** 10.3390/biology14050504

**Published:** 2025-05-05

**Authors:** Yuhan Qi, Yu Zhang, Jiali Xue, Zhen Zhang, Jingjing Cao, Nianwan Yang, Fanghao Wan, Xiaoqing Xian, Wanxue Liu

**Affiliations:** 1 State Key Laboratory for Biology of Plant Diseases and Insect Pests, Institute of Plant Protection, Chinese Academy of Agricultural Sciences, Beijing 100193, China; qyh_nwnu@163.com (Y.Q.); zhangyuu960606@163.com (Y.Z.); caojingjing319@163.com (J.C.); yangnianwan@caas.cn (N.Y.); wanfanghao@caas.cn (F.W.); 2 College of Resources and Environment, Anhui Agricultural University, Hefei 230036, China; xuejiali2024@163.com (J.X.); xjzhangzhen@163.com (Z.Z.); 3 Institute of Western Agriculture, Chinese Academy of Agricultural Sciences, Changji 831100, China

**Keywords:** potential suitable habitat, aquatic alien plant, impact factor, optimized MaxEnt

## Abstract

*Elodea nuttallii* is an ornamental macrophyte native to North America that has been introduced to Europe and Asia, and having been established, has had detrimental effects on local aquatic ecosystems. In this study, we developed an optimized MaxEnt model to predict the global potential habitat suitability for *E. nuttallii* under the influence of climate change. The model incorporated 20 relevant impact factors and occurrence record data for *E. nuttallii*. The results reveal that under current and future climate scenarios, potentially suitable habitats for *E. nuttallii* can be found on six assessed continents, mainly in Western Europe, western and eastern North America, southeastern Asia, southeastern Oceania, and scattered coastal areas in South America and Africa. Moreover, temperature and precipitation were identified as factors having significant effects on the distribution of *E. nuttallii*. In the future, the area of habitats potentially suitable for *E. nuttallii* is predicted to expand, particularly towards higher latitudes.

## Simple Summary

This study predicts the global habitat expansion of the invasive aquatic plant *Elodea nuttallii* under climate change, driven by temperature and precipitation. Suitable areas may spread to higher latitudes across six continents, threatening ecosystems.

## 1. Introduction

Freshwater plant invasion, driven by anthropogenic activities and natural dispersal, has become a severe global ecological issue. Hundreds of alien plants have been introduced into freshwater environments worldwide, causing significant adverse impacts on local biodiversity, ecological balance, and ecosystem functions [1,2,3,4]. These invasive plants, including floating, submerged, and drifting types, spread rapidly through seeds, stem fragments, and other reproductive structures, encroaching on lakes, rivers, and reservoirs [5,6]. Their proliferation not only displaces native species, but also degrades environmental conditions, such as water quality and light penetration, impairing ecosystem services [7,8].

Climate change is expected to exacerbate the problem of freshwater plant invasion. Rising temperatures and altered precipitation patterns may render previously unsuitable regions more conducive to the establishment and proliferation of invasive plants [9,10,11,12]. Additionally, changes in hydrological conditions, such as river flow and lake levels, can degrade native vegetation and create more opportunities for invasive species [13,14].

*Elodea nuttallii*, a rapidly growing submerged macrophyte native to North America, has been introduced globally and is now widely distributed in Europe and Asia [15,16,17]. It reproduces asexually and thrives in a wide range of water temperatures, making it highly adaptable [18,19]. Its invasion has led to ecological impacts like oxygen fluctuations, eutrophication, and food-web disruption, as well as economic losses due to impeded water transport and recreational activities [20,21]. Since 2005, the invasive behavior of this aquatic weed has received major attention from Asian and European countries, among which five European countries (Estonia, Poland, Spain, Switzerland, United Kingdom) list this species as a ‘Regulated Invasive Alien Plant’ and one Asian country (Jordan) classifies it as an A1 list plant [22].

Species distribution models (SDMs) are statistical tools that predict the spatial ranges and environmental adaptations of species based on environmental data and species occurrence records [23,24]. Among such models, MaxEnt is based on the principle of maximizing the information entropy, with the aim of identifying a probability distribution that is consistent with the known constraints [25]. MaxEnt offers several key advantages: it can handle both discrete and continuous data types, requires relatively small sample sizes for validation, and provides robust predictions even with limited data (as few as seven occurrence records) [26]. However, these models are prone to the risk of overfitting and have the drawback of a dependency on multiple parameters, and consequently, parameter-optimized MaxEnt models are deemed essential in determining the accuracy of prediction results [27,28].

Despite the growing awareness of the risks posed by invasive freshwater plants, current risk assessments are often insufficient. Many studies focus on local or regional scales [29,30], and there is a lack of comprehensive global assessments that account for the combined effects of climate change and human activities. Moreover, existing models often fail to capture the full range of environmental factors influencing the spread of invasive plants [31,32], leading to uncertainties in predicting future invasion risks.

In this study, we aim to narrow these gaps by predicting the global potential habitat suitability for *E. nuttallii* under current and future climate scenarios. Specifically, we focus on the following research objectives: (1) to identify the current and future global distribution of suitable habitats for *E. nuttallii* under climate change scenarios; (2) to determine the key environmental factors driving the distribution of *E. nuttallii*. We hypothesize that (1) the potential suitable habitats for *E. nuttallii* will expand significantly under future climate change scenarios, particularly towards higher latitudes; (2) temperature and precipitation will be the primary environmental factors influencing the distribution of *E. nuttallii*, with temperature playing a more dominant role. Our study will make an important contribution to understanding and regulating the dispersal of *E. nuttallii*, thereby supporting the development of freshwater environmental management plans based on ecological balance and ensuring the stable succession of aquatic biotic communities.

## 2. Materials and Methods

### 2.1. Occurrence Record Sources

**Figure 1:**
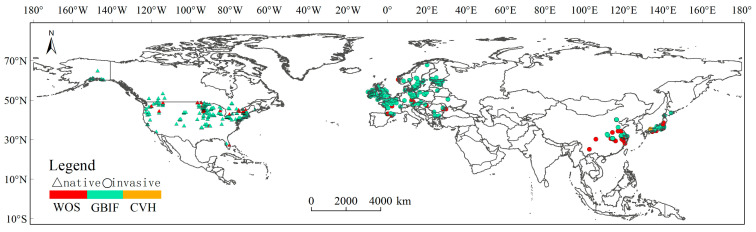
The current distribution of *Elodea nuttallii* worldwide (only invaded in the Northern Hemisphere). Note: Triangles represent native populations, circles represent invasive populations, and different colors indicate the sources of occurrence records from various databases.

Data on the presence of *E. nuttallii* were collected through distinct methods, the first of which involved searching for reported localities in the relevant open literature in the Web of Science (WOS). Secondly, we collected the latitude/longitude information for naturally occurring populations from the records of the Global Biodiversity Information Facility (GBIF) [33], and thirdly, we collected longitude and latitude information from selected specimen collection records of the Chinese Virtual Herbarium (CVH). From an initial dataset of 57,107 records, 52,731 valid occurrences remained after excluding cultivated/purchased specimens. Spatial thinning at a 5 km^2^ resolution yielded 7030 unique occurrence points, exclusively distributed across the Northern Hemisphere with 5053 (71.9%) in Europe, 1085 (15.4%) in North America, and 892 (12.7%) in Asia. Post-thinning spatial distributions showed distinct patterns across data sources—WOS contributed 135 native North American, 368 European, and 71 Asian records; GBIF provided 674 native North American, 5482 European, and 166 Asian records; while CVH contained 5 native North American, 3 European, and 126 Asian records (Figure 1).

### 2.2. Access to Impact Factors

For the purposes of the present study, as environmental factors influencing the spread of *E. nuttallii*, we considered mainly bioclimatic variables and altitude (Table 1). Data pertaining to 19 current bioclimatic factors (bio1–bio19) and an elevation factor were downloaded from the WorldClimate Database, information for which is presented in Table S1. Future bioclimatic projections were sourced from the WorldClim database [34]. For future predictions (2030s and 2050s), we used three shared socio-economic pathways (SSPs), representing future scenarios with different levels of carbon dioxide (CO_2_) concentration based on the Beijing Climate Center Climate System Model v.2—Medium Resolution model. These three scenarios (SSP1-2.6, SSP2-4.5, and SSP5-8.5) represent low, medium, and high CO_2_ concentrations, respectively [35]. On the basis of the scale of the present study, we selected a spatial resolution of 5.0 arcmin, and in cases in which we obtained Pearson correlations values |r| > 0.8 for two factors, the factor with the stronger correlation was chosen for the model. A total of nine environmental variables were ultimately retained, namely, bio2, bio5, bio11, bio12, bio14, bio15, bio18, bio19, and altitude, for the prediction of the potential suitable habitat of *E. nuttallii*.

### 2.3. Model Optimization and Precision Evaluation

To enhance the predictive accuracy of the MaxEnt model, this study focused on optimizing two critical parameters: feature combinations (L, LQ, H, LQH, LQHP) and regularization multipliers (ranging from 0.5 to 6 with incremental steps of 0.5) [36]. Using the ENMeval package (https://cran.r-project.org/web/packages/ENMeval/, accessed on 23 January 2025) in R Studio v 4.2.1 (https://www.r-project.org/, accessed on 23 January 2025), 60 parameter combinations were systematically evaluated, with model selection guided by the corrected Akaike Information Criterion (deltaAICc), where the optimal model was identified as the one with deltaAICc = 0 [37]. Leveraging 7030 georeferenced occurrence records of *E. nuttallii* and 20 environmental variables, the analysis revealed that the LQHP feature combination paired with a regularization multiplier of 0.5 achieved the highest predictive performance (Figure S1). Ten replicate simulations using these parameters yielded an average area under the receiver operating characteristic curve (AUC) of 0.821 (Figure S2), demonstrating robust reliability in mapping the species’ global potential habitats. This optimized framework effectively balances model complexity and ecological interpretability, providing a methodological advance for aquatic plant niche modeling under dynamic climatic scenarios.

### 2.4. Classification of Potential Suitable Habitats for Different Risk Levels

The ASCII-formatted output generated by the MaxEnt ecological niche model underwent raster conversion, with pixel values representing the probabilistic suitability index (*p*) for *E. nuttallii* establishment in target regions. Utilizing the maximum sensitivity-specificity threshold (cloglog threshold: *p* = 0.16) derived from receiver operating characteristic analysis, we implemented hierarchical habitat classification through the ArcGIS 10.7 spatial analyst module. The resultant risk stratification system categorizes geographic units into four ecological suitability tiers: unsuitable habitats (0 < *p* ≤ 0.16), low-suitable habitats (0.16 < *p* ≤ 0.4), medium-suitable habitats (0.4 < *p* ≤ 0.6), and high-suitable habitats (0.6 < *p* ≤ 1).

## 3. Results

### 3.1. Significant Impact Factors

**Figure 2:**
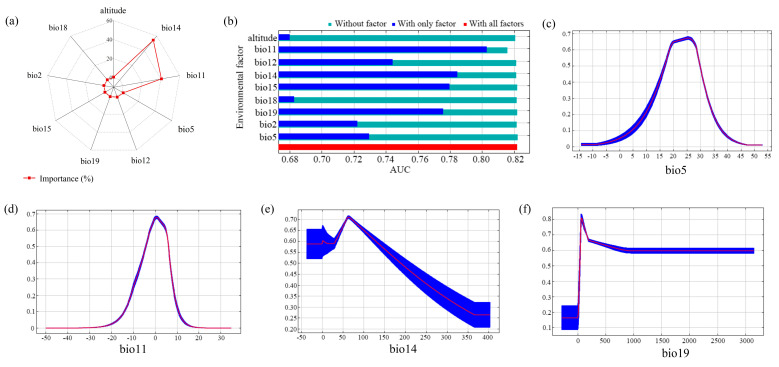
(**a**) Percentage contribution and (**b**) jackknife analysis for the key impact factors influencing the presence probability of *Elodea nuttallii*, and (**c**–**f**) response curves of the four key impact factors.

Having ranked the contributions and obtained jackknife test results for all 20 candidate impact factors, and simultaneously filtered out those factors with correlations equal to or greater than 0.8, we identified precipitation factors (bio12, bio14, bio15, bio18, and bio19), temperature factors (bio2, bio5, and bio11) and altitude as the factors most significantly associated with the potential suitable habitats of *E. nuttallii*. Notably, bio14 dominated the model contributions, reflecting its critical role in sustaining metabolic activity during drought stress, while bio11 governs the species’ overwintering capacity by regulating ice nucleation dynamics. The selected significant impact factors were modeled and analyzed in 10 replications, and the three impact factors, ranked in order of contributionm were bio14 (54.4%), bio11 (40.7%) and bio5 (1.7%) (Figure 2a). Through jackknife analysis, this study has identified dominant environmental gradient variables driving the spatial heterogeneity in *E. nuttallii*’s ecological suitability. As illustrated in Figure 2b, the bioclimatic parameters bio11 (Mean Temperature of the Coldest Quarter), bio14 (Precipitation of the Driest Month), and bio19 (Precipitation of the Coldest Quarter) exhibited marked environmental filtering effects, demonstrating significantly higher permutation importance compared to other evaluated variables. The inclusion of bio19 underscores its ecological significance in supporting root hydration during dormancy phases, a key adaptation for temperate aquatic plants.

It is generally accepted that in an environment with favorable conditions, the probability of the presence of alien plants exceeds 0.5, and consequently, by combining the response curves of key impact factors for *E. nuttallii*, we predicted that the maximum temperatures of the warmest months suitable for the survival of the species ranged from 18.5 °C to 29.1 °C (Figure 2c), a thermal window that aligns with its C3 photosynthetic pathway efficiency; the average temperatures of the coldest season ranged from −2.8 °C to −6.9 °C (Figure 2d), a range preventing lethal intracellular ice formation; the precipitation of the driest months ranged from 34.1 mm to 94.3 mm (Figure 2e), ensuring minimal water availability for rhizome survival, and the precipitation of the coldest season ranged from 131.4 mm to 415.6 mm (Figure 2f), a threshold maintaining sediment moisture for propagule persistence.

### 3.2. Globally Potential Suitable Habitats Currently and in the Future

**Figure 3:**
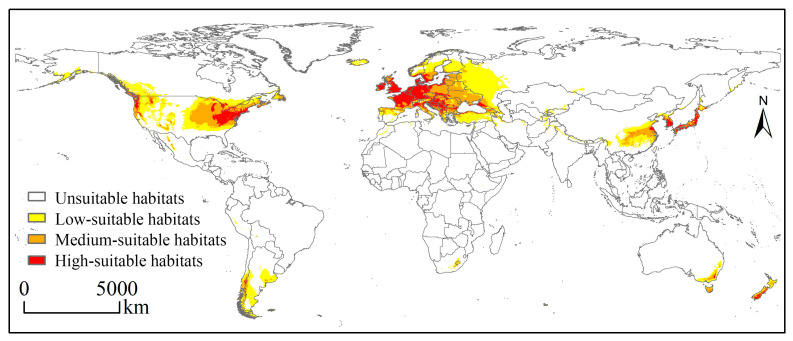
Potentially suitable habitats for *Elodea nuttallii* globally under the current climate scenario.

**Figure 4:**
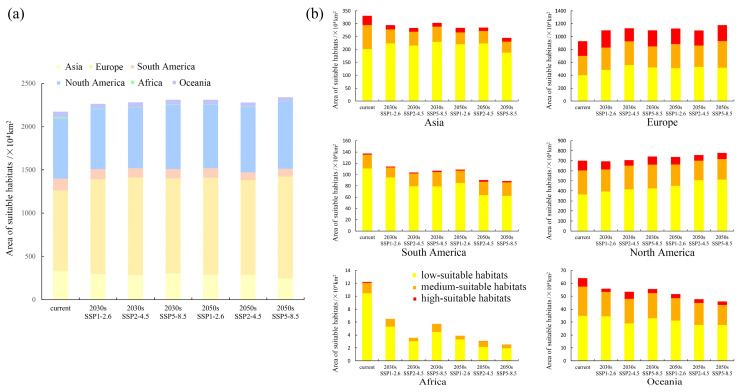
(**a**) Areas of suitable habitats for *Elodea nuttallii* in six continents under different climate scenarios; and (**b**) areas of different classes of suitable habitats for *E. nuttallii* in six continents under different climate scenarios.

The model predictions under current climatic conditions reveal that globally, the range of potentially suitable habitats for *E. nuttallii* is wider than that indicated by current authenticated occurrence records, with suitable habitats on six continents (Figure 3), and an overall area of 2174.34 × 10^4^ km^2^ of habitats potentially suitable for its growth (Figure 4a). Among these, Europe was identified as the continent with the largest area of suitable habitats and highly suitable habitats for *E. nuttallii,* with a suitable habitat area of 930.11 × 10^4^ km^2^, accounting for approximately 42.78% of the global total suitable habitat area (Figure 4a), and 227.89 × 10^4^ km^2^ of highly suitable habitat area (Figure 4b). Comparatively, in North America, an area of 699.53 × 10^4^ km^2^ was deemed suitable habitat for *E. nuttallii*, accounting for approximately 32.17% of the global total suitable habitat area, whereas in Asia, the area of suitable habitats was 330.84 × 10^4^ km^2^, accounting for approximately 15.22% of the global total (Figure 4a). In contrast, South America, Oceania, and Africa would appear to have a few areas with habitats suitable for *E. nuttallii* colonization (Figure 3), with Africa containing only low- and medium-suitable habitats and a narrow range of relatively unsuitable habitats for *E. nuttallii*. From different geographical and administrative perspectives, the area of highly suitable habitat for *E. nuttallii* was smaller and more aggregated, and mainly concentrated along western coastal areas of Western European countries (United Kingdom, Germany, Denmark, Netherlands, Belgium, France, Germany, and Italy), a few areas of North America, the eastern coasts of Asian countries (China, South Korea, and Japan), the southern parts of South America (Chile), and the southeastern parts of Oceania (Australia and New Zealand). The medium-suitable habitats covered a large area, which was mainly distributed in Eastern Europe and eastern North America, with sporadic distribution in eastern Asia (mainly China), southern South America, southwestern Oceania, and southern Africa. The low-suitable habitats covered the widest range, mainly concentrated in Western Europe, North America, and eastern Asia. A further finding worth noting is that a number of coastal areas in the Southern Hemisphere are also potentially suitable for *E. nuttallii* (Figure 3).

**Figure 5:**
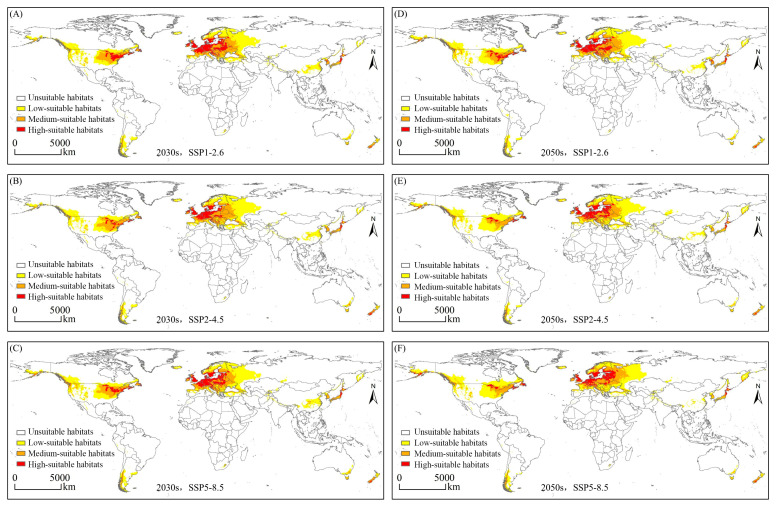
Potentially suitable habitats for *Elodea nuttallii* globally under future climate scenarios.

Using three shared socio-economic pathway scenarios based on future climate data, we sought to predict the distribution of habitats that would be suitable for *E. nuttallii* in the 2030s and 2050s. Notably, compared with our findings for the distribution of suitable habitats under current climate conditions, we detected an increasing trend of expansion in the total habitat area suitable for *E. nuttallii* under future climate scenarios, with small variations in the extent of the distribution of suitable habitats in each class, which typically manifested as a small variation in the marginal zones of suitable habitats (Figure 4 and Figure 5). With the exception of the SSP2-4.5 scenario, in which the area of suitable habitats remained essentially constant between the 2030s and 2050s, the area of suitable habitats in the 2050s was greater than that in the 2030s under the other two assessed scenarios, with the greatest increase occurring under the SSP5-8.5 scenario (Figure 4). From different geographical and administrative perspectives, with the exception of a slight reduction in the 2030s, suitable habitats for *E. nuttallii* in North America expanded outward. Under the SSP1-2.6 scenario, however, there were predicted reductions in suitable habitats in Asia, South America, Africa, and Oceania. In particular, in China, we observed a significant contraction in suitable habitat area, along with a clear decline in the extent of medium-suitable habitats and a virtual absence of highly suitable habitats (Figure 5).

### 3.3. Variations in the Spatial Distribution of Suitable Habitats

**Figure 6:**
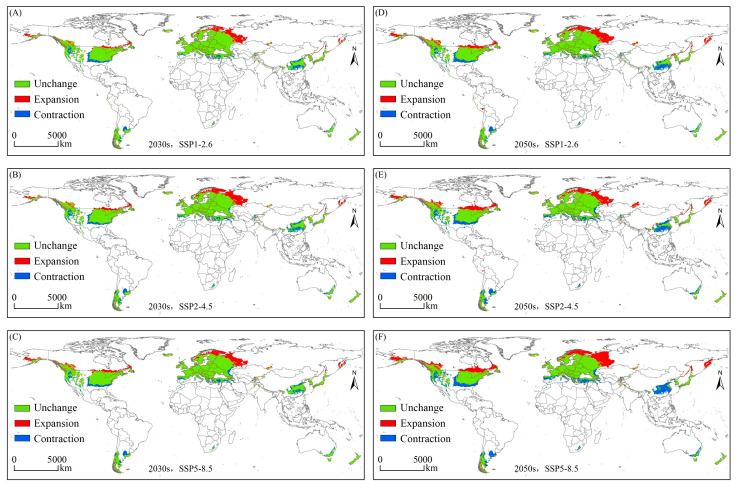
Spatial variations of potentially suitable habitats for *Elodea nuttallii* globally under future climate conditions.

Under future climate scenarios, we observed a significant variation in the spatial distribution of suitable habitats for *E. nuttallii*, with contractions of suitable habitats in Asia, South America, North America, Africa, and Oceania, and with that in Asia being the most pronounced. Contrastingly, there were predictions of different extents of suitable habitat expansion in Europe, South America, and Asia, with notably significant expansions of suitable habitats in Europe, covering almost the entire area of the European continent after the diversification of suitable habitats (Figure 6). This would thus tend to indicate that in the future, the climate in Europe could be very favorable for the colonization of *E. nuttallii*. Although our simulations predicted contractions in habitat suitability in some regions, overall, it appears that there will be a spatial expansion of suitable habitats for *E. nuttallii* in the medium-term (Table 2).

**Figure 7:**
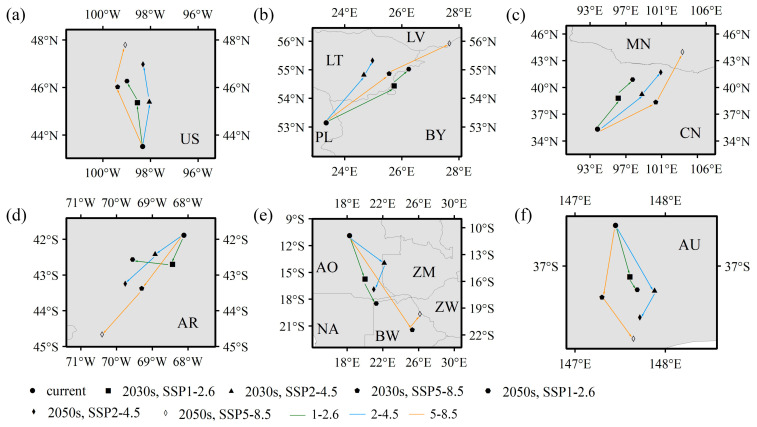
Future centroid shifts of *Elodea nuttallii* habitats in (**a**) North America, (**b**) Europe, (**c**) Asia, (**d**) South America, (**e**) Africa, and (**f**) Oceania under different climate scenarios (US, United States; PL, Poland; LT, Lithuania; LV, Latvia; BY, Belarus; MN, Mongolia; CN, China; AR, Argentina; AO, Angola; NA, Namibia; BW, Botswana; ZM, Namibia; ZW, Zimbabwe; AU, Australia).

We subsequently analyzed variations in climate-mediated centroids that can reflect spatial variations, mainly latitudinal and longitudinal, in potentially suitable habitats for *E. nuttallii* (Figure 7). For the six assessed continents, using ArcGIS, we obtained the geographical centroids of suitable habitats of *E. nuttallii* for each climate scenario, and accordingly detected certain trends. Notably, variations in the centroids of suitable habitats under future climate scenarios were generally consistent; i.e., suitable habitats for *E. nuttallii* consistently shifted to higher latitudes. This poleward migration necessitates enhanced biosecurity surveillance in northern regions, particularly through early detection networks targeting river corridors and wetlands vulnerable to new colonization. In terms of the extent of these shifts, the centroids of suitable habitats in North America, South America, and Oceania did not extend beyond the administrative boundaries of any single country (the United States, Argentina, or Australia), although spatial shifts were observed. For these nations, adaptive management should prioritize updating invasive species risk maps and strengthening domestic quarantine protocols around shifting hotspots. Contrastingly, in Europe and Africa, the centroids of suitable habitats for *E. nuttallii* involved two countries under scenarios SSP1-2.6 and SSP2-4.5 and three countries under scenario SSP5-8.5. Such transboundary shifts demand bilateral/multilateral agreements for coordinated eradication efforts, particularly in shared watersheds. In Asia, there were no shifts in the centroid of suitable habitats involving more than two countries (China and Mongolia) under any of the future climate scenarios. This limited cross-border expansion highlights the urgency for Sino-Mongolian joint action plans, including harmonized herbicide application standards and real-time invasion data sharing platforms.

## 4. Discussion

On account of its rapid reproduction and competitive ability, *E. nuttallii* has been described as an invasive macrophyte of concern. However, whereas to date, studies have focused primarily on potentially suitable habitats in localized regions of European countries [38], this approach aligns with broader ecological modeling efforts, as other invasive freshwater plants such as *Eichhornia crassipes* (water hyacinth) and *Myriophyllum spicatum* (Eurasian watermilfoil) have also been successfully predicted for their suitable distributions using species distribution models like MaxEnt, demonstrating high spatial accuracy in identifying invasion-prone zones [39,40]. This aquatic plant is also becoming a damaging invasive species in Asia, and even globally. In this study, we performed the first assessment of potential trends in the global geographical distribution of *E. nuttallii* in response to the influence of climate change.

### 4.1. Influence of Impact Factors on Potentially Suitable Habitats

Habitats potentially suitable for *E. nuttallii* globally were found to be synergistically influenced by multiple environmental factors of temperature, precipitation, and altitude, with temperature and precipitation making the most significant contributions (bio11, bio14, et al.). Our findings in this study reveal that the growth environment for *E. nuttallii* tended to become unsuitable when the maximum temperature of the warmest month was approximately 29 °C or above, and the average temperature of the coldest quarter was approximately −7 °C or less, which is consistent with the findings of previous studies indicating that *E. nuttallii* typically thrives in sunny but cooler freshwater habitats, with optimal growth temperatures ranging from 10 to 25 °C [41]. Within the range of suitable growth temperatures, an increase in temperature generally promotes the accelerated growth and reproduction of aquatic alien plants, as has previously been observed in the case of *E. nuttallii* [42], thereby providing competitive advantage, and consequently an inhibition of native plant growth. Moreover, given its high ecological resilience (i.e., low-temperature tolerance) [43], *E. nuttallii* can thrive in areas characterized by significant temperature variability between highs and lows, such as those experienced in Europe, and may thus have a higher reproductive potential than the native flora, which would accordingly be conducive to establishing populations in new environments, with subsequent rapid expansion.

Precipitation is an essential factor contributing to the growth and reproduction of aquatic plants, and it has been established that *E. nuttallii* is dependent on levels of precipitation exceeding approximately 34 mm in the driest month, and 131 mm in the coldest season. Higher levels of precipitation may enhance the water-use efficiency of *E. nuttallii*, thereby accelerating the rate of growth and reproduction, and thereby enhancing the likelihood of successfully establishing populations in newly colonized areas [44]. Moreover, an increase in precipitation can potentially contribute to an expansion of the range of habitats suitable for the colonization of aquatic alien plants, thereby facilitating adaptation to wider environments [45] and the establishment of populations in areas that would otherwise be inhospitable. In addition, compared with bioclimatic factors, our finding that altitude makes only a relatively small contribution to the distribution of *E. nuttallii* may indicate that, given suitable climatic conditions, this plant is generally well adapted to a moderate range of elevations in watershed environments in tropical, subtropical or temperate regions.

### 4.2. Variations in Potentially Suitable Habitat for Elodea nuttallii

Predicting the potential global distribution of *E. nuttallii* under conditions of future climate change can contribute to identifying probable invasion hotspots. *Elodea nuttallii* has a wide potential distribution beyond its currently established distribution in North America, Europe, and Asia, thereby highlighting the potential risk of invasion in regions lying within mid-latitudes of the Southern Hemisphere. According to the Köppen climate classification of global climate zones [46], the coastal regions of southern Chile, southern Argentina, south-eastern Australia, and New Zealand have a climate type similar to the temperate marine climate regions of the Northern Hemisphere, particularly with respect to temperature and precipitation, among which *E. nuttallii* has invaded extensive areas of some European countries such as Norway and Sweden. Furthermore, the southern part of South Africa has a Mediterranean climate type, similar to that of countries such as Spain, Portugal, and Italy in the southern and western parts of Europe. Consequently, even though the aforementioned countries in the Southern Hemisphere have yet to be colonized by *E. nuttallii*, they remain at a potentially high risk of future invasion.

Our model-based predictions indicate the likelihood that the area of habitat potentially suitable for *E. nuttallii* colonization will gradually expand in the mid-term future, particularly by the 2050s under the SSP5-8.5 scenario of elevated temperatures, large-scale population growth, and continued high CO_2_ emissions. In this regard, it is hypothesized that three sequences of events could contribute to an expansion of suitable habitats. The first posits that an increase in temperature could lead to a modification in precipitation patterns [47], in response to which a larger number of regions will have temperature and humidity conditions that reach the thresholds for the growth and reproduction of *E. nuttallii*. In the second scenario, under conditions of continued high CO_2_ emissions, changes in population growth and urbanization may contribute to altered land-use patterns and an increase in the amount and area of suitable habitats for *E. nuttallii*, such as waterbodies and wetlands [48]. The third assumption is that international trade and transport will become more frequent and developed, thereby increasing the number of pathways and opportunities for the introduction and dispersal of *E. nuttallii* [49], and thus the likelihood of survival and colonization. However, these projections are constrained by key model limitations: (1) dispersal barriers (e.g., mountain ranges, hydrological discontinuities) are not quantified [50]; (2) biotic resistance from native competitors (e.g., *Vallisneria natans*) is omitted [51]; and (3) anthropogenic filters such as targeted eradication policies or trade restrictions remain unmodelled [52].

Consistent with the projected migration patterns of the aquatic macrophyte *E. nuttallii* described herein, numerous previous studies have predicted the migration of invasive alien plants to higher latitudes in response to future climate change [53]. As global temperatures continue to rise, the climate in previously cooler high-latitudes regions is gradually warming. Such changes have accordingly contributed to an expansion in the ecological niche of *E. nuttallii*, with temperature and humidity conditions at certain high latitudes now considered favorable for the growth and reproduction of this species. Moreover, climate change-induced extreme weather events and natural disasters, including floods, droughts, and forest fires, may lead to greater perturbations of ecosystems at higher latitudes [54]. Such environmental variation may provide *E. nuttallii* with survival opportunities, thereby facilitating the endurance and reproduction of this plant at higher latitudes.

### 4.3. Early Warning Recommendations

For countries that are suitable for the growth and reproduction of *E. nuttallii*, the establishment of relevant laws and regulations to enhance border security is essential, especially since most developing countries currently lack comprehensive legislation targeting invasive species [55]. The content of such legislation should encompass detailed aspects of invasiveness prediction, risk assessment, and emergency management [56]. Invasive aquatic plants like *E. nuttallii* often cross national borders, and consequently, transnational cooperation and joint efforts are required to address the mutual threat. A unified invasion forecasting platform could be developed using ensemble modeling approaches, integrating climate scenarios, trade network analysis, and species distribution models to simulate transboundary spread patterns under different policy interventions [57]. This system would enable synchronized border controls and resource allocation across neighboring nations. Moreover, detailed protocols for the construction and operation of aquaculture facilities should be established by each country. In many developing countries, aquaculture is managed by individual farmers, and measures to prevent the escape of invasive aquatic species are inadequate. To prevent accidental spread, regular improvements should be made to aquaculture structures (e.g., nets to intercept invasive plants, deeper ponds) and unified inspection and management measures should be implemented [58]. Most importantly, global efforts should be intensified to raise awareness and provide specific tools for the prevention and control of invasive species. Ensuring the smooth implementation of each of these measures will greatly protect global biosecurity and human well-being.

## 5. Conclusions

The optimized MaxEnt model developed in this study was established to have good precision in predicting the extent of habitats potentially suitable for the invasive macrophyte *E. nuttallii*, using which we predicted that climate change will influence the future distribution and potential spread of this plant. It should be noted that realized invasion outcomes depend not only on climatic suitability, but also on propagule pressure from human activities and biotic resistance from native plant communities. Under current and future climate scenarios, suitable habitats for *E. nuttallii*, particularly highly suitable habitats, were predominantly distributed in the northern temperate zone, and to a lesser extent in the southern temperate zone. Regardless of the assessed scenario (SSP1-2.6, SSP2-4.5, or SSP5-8.5), modeling indicted that the range of habitats potentially suitable for *E. nuttallii* will expand by the 2030s and 2050s, with the most pronounced expansion being predicted in the 2050s under the SSP5-8.5 scenario. In response to on-going global warming, expansion will tend to be more evident at higher latitudes. Among the environmental factors assessed, temperature, precipitation, and elevation were identified as important factors influencing the potential suitability of habitats for *E. nuttallii*.

## Figures and Tables

**Table 1:** The 20 environmental factors and their abbreviations and full names.

Abbreviations	Full Names	Abbreviations	Full Names
bio 1	Annual Mean Temperature	bio 11	Mean Temperature of Coldest Quarter
bio 2	Mean Diurnal Range	bio 12	Annual Precipitation
bio 3	Isothermality	bio 13	Precipitation of Wettest Month
bio 4	Temperature Seasonality	bio 14	Precipitation of Driest Month
bio 5	Max Temperature of Warmest Month	bio 15	Precipitation Seasonality
bio 6	Min Temperature of Coldest Month	bio 16	Precipitation of Wettest Quarter
bio 7	Temperature Annual Range	bio 17	Precipitation of Driest Quarter
bio 8	Mean Temperature of Wettest Quarter	bio 18	Precipitation of Warmest Quarter
bio 9	Mean Temperature of Driest Quarter	bio 19	Precipitation of Coldest Quarter
bio 10	Mean Temperature of Warmest Quarter	Altitude	Altitude

**Table 2:** Variations in the areas and proportions of potentially suitable habitats under future climate conditions—expansion.

	Indicators	2030s			2050s		
		SSP1-2.6	SSP2-4.5	SSP5-8.5	SSP1-2.6	SSP2-4.5	SSP5-8.5
Expansion	Area/×10^4^ km^2^	331.26	392.13	413.24	465.19	513.54	656.28
	Proportion/%	2.17	2.57	2.71	3.05	3.36	4.30
Contraction	Area/×10^4^ km^2^	242.27	286.89	275.83	327.89	409.09	490.01
	Proportion/%	1.59	1.88	1.81	2.15	2.68	3.21
Unchange	Area/×10^4^ km^2^	1939.51	1894.90	1905.96	1853.90	1772.70	1691.77
	Proportion/%	12.70	12.41	12.48	12.14	11.61	11.08

## Data Availability

The data cannot be shared due to privacy concerns.

## Supplementary Materials

The following supporting information can be downloaded at: https://www.mdpi.com/article/10.3390/biology14050504/s1, Figure S1: Optimal parameter combinations of the MaxEnt model (L, linear; Q, quadratic; H, hinge; P, product); Figure S2: The mean AUC values for the optimised MaxEnt model (AUC, the area under receiver operating characteristic (ROC) curve); Table S1: the 20 impact factors and their categories, abbreviations, and full names.
