# Supplementary material for: Future Climate Change Increases the Risk of Suitable Habitats for the Invasive Macrophyte Elodea nuttallii
Source: Biology (Basel). 2025 May 5;14(5):504. doi: 10.3390/biology14050504 (PMC12109238; doi:10.3390/biology14050504)
Supplement: Supplementary file 1 [file biology-14-00504-s001.zip › biology-3604042-supplementary.pdf]

Supplementary File

Tab. S1 the 20 impact factors and their categories, abbreviations, and full names

Category	Abbreviation	Full name
	bio1	Annual Mean Temperature
	bio2	Mean Diurnal Range (Mean of monthly (max temp - min temp))
	bio3	Isothermality (bio2/bio7) (×100)
	bio4	Temperature Seasonality (standard deviation ×100)
	bio5	Max Temperature of Warmest Month
	bio6	Min Temperature of Coldest Month
	bio7	Temperature Annual Range (bio5-bio6)
	bio8	Mean Temperature of Wettest Quarter
	bio9	Mean Temperature of Driest Quarter
bioclimatic	bio10	Mean Temperature of Warmest Quarter
	bio11	Mean Temperature of Coldest Quarter
	bio12	Annual Precipitation
	bio13	Precipitation of Wettest Month
	bio14	Precipitation of Driest Month
	bio15	Precipitation Seasonality (Coefficient of Variation)
	bio16	Precipitation of Wettest Quarter
	bio17	Precipitation of Driest Quarter
	bio18	Precipitation of Warmest Quarter
	bio19	Precipitation of Coldest Quarter
topographical	altitude	Altitude

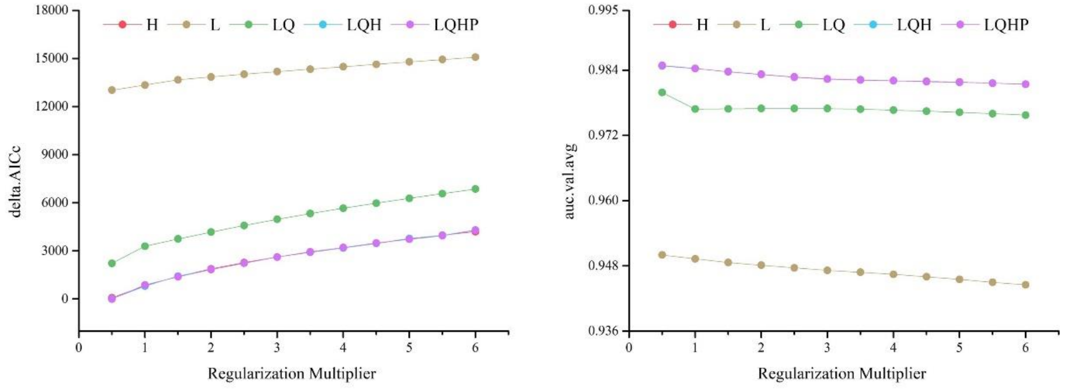

Fig. S1 Optimal parameter combinations of the MaxEnt model (L: linear; Q: quadratic; H: hinge; P: product)

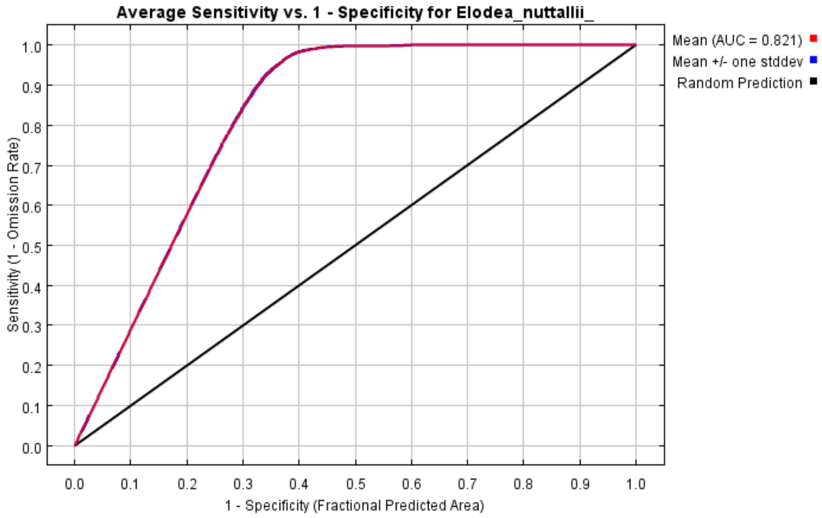

Fig. S2 The mean AUC values for the optimised MaxEnt model (AUC: the area under receiver operating characteristic (ROC) curve)
